# Identification of ambiguities in the 1994 chronic fatigue syndrome research case definition and recommendations for resolution

**DOI:** 10.1186/1472-6963-5-37

**Published:** 2005-05-13

**Authors:** Bart Stouten

**Affiliations:** 1 Violierstraat 27, 5402 LA Uden, The Netherlands

## Abstract

**Background:**

A recent article by Reeves *et al*. on the identification and resolution of ambiguities in the 1994 chronic fatigue syndrome (CFS) research case definition recommended the Checklist Individual Strength, the Chalder Fatigue Scale, and the Krupp Fatigue Severity Scale for evaluating fatigue in CFS studies. To be able to discriminate between various levels of severe fatigue, extreme scoring on the individual items of these questionnaires must not occur too often.

**Methods:**

We derived an expression that allows us to compute a lower bound for the number of items with the maximum item score for a given study from the reported mean scale score, the number of reported subjects, and the properties of the fatigue rating scale. Several CFS studies that used the recommended fatigue rating scales were selected from literature and analyzed to verify whether abundant extreme scoring had occurred.

**Results:**

Extreme scoring occurred on a large number of the items for all three recommended fatigue rating scales across several studies. The percentage of items with the maximum score exceeded 40% in several cases. The amount of extreme scoring for a certain scale varied from one study to another, which suggests heterogeneity in the selected subjects across studies.

**Conclusion:**

Because all three instruments easily reach the extreme ends of their scales on a large number of the individual items, they do not accurately represent the severe fatigue that is characteristic for CFS. This should lead to serious questions about the validity and suitability of the Checklist Individual Strength, the Chalder Fatigue Scale, and the Krupp Fatigue Severity Scale for evaluating fatigue in CFS research.

## Text

Since ambiguities in the 1994 chronic fatigue syndrome (CFS) research case definition [1] do indeed contribute to inconsistenties in the identification of cases, I welcome the publication by Reeves *et al. *[2] and the authors' efforts to resolve these problems. However, I have to express my deepest concerns about the three instruments that the authors have recommend for measuring fatigue in research studies on CFS. Because all three instruments easily reach the extreme ends of their scales on a large number of the individual items, they do not accurately represent the severe fatigue that is required to satisfy any of the published CFS research case definitions [1,3-5]. This low ceiling effect seriously distorts the fatigue measurements, which will inevitably result in bias and potentially misleading results.

To verify that the three recommended instruments do indeed exhibit low ceiling effects, one can study the mean scale scores that are reported in the literature. The recommended instruments were the Checklist Individual Strength (CIS) [6], the Chalder Fatigue Scale [7], and the Krupp Fatigue Severity Scale [8]. Each of these questionnaires consists of a fixed number of questions or statements. The answer to each question or the degree to which the participant agrees with a statement is scored on a certain scale. A question or statement with its corresponding scale is referred to as an *item*, and the assigned value corresponding to the participant's answer as the *item score*. A participant's fatigue rating scale score *Y *is computed by summing his individual item scores.

We can derive a lower bound *L *for the number of items with a maximum score for a given study by combining the reported mean fatigue rating scale score with the properties of the scale. Let us denote the reported number of subjects by *n *and the mean scale score of these subjects by . We consider instruments that consist of *N *items, with *m *possible scores for each item. Each item score is an element of the set {*S*_1_, *S*_2_,..., *S*_*m *- 1_, *S*_*m*_}, where *S*_*i *- 1 _<*S*_*i*_. Hence, *S*_1 _and *S*_*m *_are respectively the minimum and maximum possible item scores. We count the number of items with a certain score *S*_*i*_, and denote this number by *k*_*i*_. Because we have *n *individuals who each answered *N *questions, the *k*_*i*_'s add up to *nN*. Consequently,



The sum of the item scores of all individuals together is equal to *n*. Moreover, it is also equal to . Since *S*_*i *- 1 _<*S*_*i*_, we find that



Hence, we find that the lower bound *L *that we were looking for is given by



If *L *should be negative, which happens when  is less than *N S*_*m *- 1_, then we set *L *to zero. A lower bound for the percentage of items with the maximum score is . Note that this percentage is independent of the number of subjects in the study.

Lower bounds *L *for the number of items with the maximum score corresponding to data reported in literature were computed for each of the recommended fatigue rating scales. Because a recent Dutch article [9] recommended the Shortened Fatigue Questionnaire (SFQ) for assessing fatigue in clinical practice, this scale was also included in the analysis. The SFQ is simply a reduced version of the CIS 'fatigue severity' subscale, so the two are closely related.

At least two articles per fatigue rating scale were selected on a rather arbitrary basis. Subjects fulfilled the CDC88-CFS [3], Oxford-CFS [5], CDC94-CFS [1], or CDC94-UCF (unexplained chronic fatigue, i.e. either CFS or idiopathic chronic fatigue) [1] criteria. In particular, the study by Vercoulen *et al. *[10] was selected because it contains detailed data on the distribution of the scores for each CIS subscale. The study by Alberts *et al. *[11] was included because it contained normative data for the SFQ. The study by Vermeulen *et al. *[12] was selected to also include data on the SFQ from another source than the University Medical Centre Nijmegen. The article by Jason *et al. *[13] was selected because it was specifically concerned with the reliability and validity of a screening instrument for CFS. A recent Cochrane review [14] has investigated the relative effectiveness of exercise therapy and control treatments for CFS. All four studies that were included in that review and that have already been published [15-18] were analyzed here (one study by Moss-Morris *et al. *that was included in the review was submitted but not yet published). The other studies were selected because they were easily available to the author. Baseline data for Friedberg and Krupp [19] and Deale *et al*. [20] were read from the graphs presented in the articles. It is remarked that the 'matched ambulant group' in Van der Werf *et al*. [21] is a subset of the 'total ambulant group' in that study. Furthermore, the 'research participants' in Van der Werf *et al. *[22] are the same subjects as the 'total ambulant group' in [21].

The lower bounds for the number of items with the maximum score are presented in Table 1. From the lower bounds listed in the last column of the table we see that for several studies the number of items with the maximum score is larger than 40%. It is emphasized that the lower bounds were derived assuming a worse case scenario for the distribution of the item scores, i.e. participants have either the highest or the second highest possible score on each item. Since the worse case distribution is quite unrealistic, in reality the percentages of items with the maximum score are generally (even) higher than the values reported in the table. For example, according to the table it is not possible to conclude that extreme scoring occurred on the 'physical activity' subscale of the CIS in the study by Vercoulen *et al. *[10]. However, according to additional data listed in that article the 80th percentile of the 'physical activity' subscale is equal to the maximum possible subscale score of 3 × 7 = 21. Thus approximately 20% of the subjects reached the extreme score on all of their items, from which we can infer that extreme scoring occurred on at least 20% of the items.

**Table 1:** Lower bounds for the number of items with the maximum score for several studies. *N *is the number of items that constitute the (sub)scale, *S*_*m *_is the maximum possible individual item score, *n *is the reported number of subjects,  is the reported mean (sub)scale score, and *L *is the derived lower bound for the number of items with the maximum score. The last column lists a lower bound for the percentage of items with the maximum score based on *L*. The second highest possible item score *S*_*m *- 1_ is equal to *S*_*m *- _1 for all considered (sub)scales.

Scale	*N*	*S*_*m*_	*n*		*L*	
Checklist Individual Strength	Oxford-CFS, CDC94-UCF; Vercoulen *et al. *[10]					

-fatigue severity subscale	8	7	758	51.7	2805	**46%**
-physical activity subscale	3	7	758	16.9	0	**0%**
-reduced motivation subscale	4	7	758	17.0	0	**0%**
-concentration subscale	5	7	758	27.5	0	**0%**

Checklist Individual Strength	CDC94-UCF; van der Werf *et al. *[21]					

-homebound group; fatigue severity subscale	8	7	18	53.6	101	**70%**
-matched ambulant group; fatigue severity subscale	8	7	32	52.8	154	**60%**
-total ambulant group; fatigue severity subscale	8	7	270	52.1	1107	**51%**
-homebound group; physical activity subscale	3	7	18	15.8	0	**0%**
-matched ambulant group; physical activity subscale	3	7	32	17.0	0	**0%**
-total ambulant group; physical activity subscale	3	7	270	17.6	0	**0%**
-homebound group; concentration subscale	5	7	15	22.4	0	**0%**

Shortened Fatigue Questionnaire	van der Werf *et al. *[22]					

-survey respondents (Dutch ME-Association members)	4	7	1955	23.9	0	**0%**
-research participants (CDC94-UCF)	4	7	270	26.1	567	**53%**

Shortened Fatigue Questionnaire	Oxford-CFS, CDC94-UCF; Alberts *et al. *[11]					

-normative data for CFS	4	7	445	26 to 27	890	**50%**

Shortened Fatigue Questionnaire	CDC94-CFS; Vermeulen *et al. *[12]					
-study group	4	7	35	24.8	28	**20%**

Krupp Fatigue Severity Scale	CDC88-CFS; Friedberg *et al. *[19]					

-treatment group	9	7	22	58	88	**44%**
-no-treatment group	9	7	22	51	0	**0%**

Krupp Fatigue Severity Scale	CDC88-CFS; DeLuca *et al. *[23]					

-subjects with concurrent axis 1 psychiatric disorder	9	7	12	58.5	54	**50%**
-subjects without concurrent psychiatric disorder	9	7	21	57.2	67	**36%**

14-item Chalder Fatigue Scale	Oxford-CFS; Wearden *et al. *[15]					

-'exercise and fluoxetine group'	14	3	33	35.9	261	**56%**
-'exercise and placebo group'	14	3	34	33.7	194	**41%**
-'exercise control and fluoxetine group'	14	3	35	34.4	224	**46%**
-'exercise control and placebo group'	14	3	34	34.0	204	**43%**

14-item Chalder Fatigue Scale	Oxford-CFS; Fulcher *et al. *[16]					

-exercise group	14	3	33	28.9	30	**6%**
-fiexibility group	14	3	33	30.5	83	**18%**

11-item Chalder Fatigue Scale	CDC94-CFS; Jason *et al. *[13]					

-physical subscale	7	3	15	18.40	66	**63%**
-mental subscale	4	3	15	9.13	17	**28%**

11-item Chalder Fatigue Scale	CDC94-CFS; Wallman *et al. *[17]					

-exercise group; physical subscale	7	3	32	11.6	0	**0%**
-exercise group; mental subscale	4	3	32	6.3	0	**0%**
-relaxation/flexibility group; physical subscale	7	3	29	11.4	0	**0%**
-relaxation/flexibility group; mental subscale	4	3	29	5.6	0	**0%**

11-item bimodal Chalder Fatigue Scale	Oxford-CFS and CDC94-CFS; Deale *et al. *[20]					

-cognitive behavior therapy group	11	1	30	10.1	303	**92%**
-relaxation group	11	1	30	9.3	279	**85%**

11-item bimodal Chalder Fatigue Scale	Oxford-CFS; Powell *et al. *[18]					

-control group	11	1	34	10.6	360	**96%**
-minimum intervention group	11	1	37	10.4	385	**95%**
-telephone intervention group	11	1	39	9.9	386	**90%**
-maximum intervention group	11	1	38	10.2	388	**93%**

It should be clear that extreme scoring on a large number of items occurred for all scales across several studies. Only the 'concentration' and 'reduced motivation' subscales of the CIS did not show evidence of extreme scoring. That the amount of extreme scoring for a certain scale varies from one study to another suggests heterogeneity in the selected subjects across studies. Since the studies that were analyzed were selected on a rather arbitrary basis and not in a systematic way, the data in Table 1 should not be regarded as a true reflection of the CFS literature as a whole. The main point is that it does prove that abundant extreme scoring occurred for all the recommended fatigue rating scales in at least some of the CFS studies published in literature.

One only needs to glance at the three recommended instruments to understand why extreme scoring occurs so often. The CIS and the Krupp Fatigue Severity Scale consist of statements like "I feel tired" and "I am easily fatigued" that are scored on seven-point scales (from "yes, that is true" to "no, that is not true" for the CIS; from "strongly disagree" to "strongly agree" for the Krupp scale). Thus it does not matter whether a subject feels 'extremely tired,' 'severely tired' or 'just tired,' and is 'easily extremely fatigued,' 'easily severely fatigued' or 'easily fatigued;' he will score on the extreme end of the scale for all these cases. A similar argument applies to the Chalder Fatigue Scale, where the participant has to choose from one of four answers like "less than usual," "no more than usual," "more than usual" and "much more than usual" to questions such as "Do you feel weak?" For the continuous version of the Chalder scale answers are rated from 0 to 3, for the bimodal version the scoring system is {0, 0, 1, 1}. This explains why the binary version performs even worse than the continuous version.

Interestingly, the ceiling effect has been noted before by members of the International CFS Study Group in their individual publications: "The CIS-fatigue score [i.e. the 'fatigue severity' subscale of the CIS] involves an overall rating and in CFS samples easily reaches the extreme end of its scale" [21]; "a ceiling effect in the [Krupp] Fatigue Severity Scale may limit its utility to assess severe fatigue-related disability" [24]. A publication that examined the distribution of the 14 items of the Chalder Fatigue Scale in 136 CFS patients found that "Scores on eight items were normally distributed, but six items ('tiredness,' 'resting more,' 'lacking energy,' 'feeling weak,' 'feeling sleepy or drowsy,' and 'starts things without difficulty but gets weaker as goes on') were highly skewed with the majority of patients reaching the maximum score" [25].

Abundant extreme scoring and the corresponding inability to discriminate between various levels of severe fatigue can lead to misleading results in several ways. For example, van der Werf *et al*. [21] compared a group of 18 homebound CF(S) patients with a group of 32 matched ambulant CF(S) patients. No significant difference was found when fatigue was measured with the CIS 'fatigue severity' subscale (*p *= 0.39). But when fatigue was measured with the 'Daily Observed Fatigue' scale that does not exhibit such a strong ceiling effect, it was concluded that the homebound group was significantly more fatigued than the ambulant group (*p *< 0.01). Another problem occurs when studying the relation between the experienced level of fatigue and another factor such as social support. Then the correlation between the two will certainly be distorted if the fatigue measurement has a low ceiling effect and the other measure has not. The most dangerous situation however arises when a scale with low ceiling is used as a primary outcome measure to evaluate a CFS treatment. Consider five patients with a baseline CIS-fatigue score of 52 (e.g. the mean baseline score in Prins *et al*. [26] was 52.1). Suppose one patient improves (e.g. CIS-fatigue = 16 at follow-up) and the other four patients become extremely fatigued due to treatment (CIS-fatigue = 56 at follow-up, i.e. the maximum scale score). Then still the overall mean has improved from 52 to 48, even though 80% of the subjects are substantially more fatigued after treatment. In particular, participants who already have the maximum scale score at baseline can never get worse according to the 'recommended' fatigue rating scales. Systematic errors that may result in artificial treatment effects opposite to the true situation should be avoided at all times.

Unfortunately, the reasons for recommending the CIS, the Krupp and the Chalder scales in the main article text are limited to 'they have been used before,' 'normative data have been collected' and 'receiver-operating characteristics have been published.' In the Author's response to reviews (25 July 2003) that is available on the pre-publication site of the article, the authors remark that these are all 'standardized, validated, internationally accepted instruments' without giving any reference to support this statement. Although the recommended fatigue rating scales might indeed be accepted by numerous scientists of various nationalities, the evidence presented here must lead to serious questions about their validity and suitability for CFS research.

Noticeably, the Profile of Fatigue-Related Symptoms (PFRS) that was developed more than a decade ago by Ray *et al. *[27,28] is a rating scale that does not has the flaw of low ceiling in CFS samples. It consists of the four subscales 'Emotional Distress,' 'Cognitive Difficulty,' 'Fatigue' and 'Somatic Symptoms.' All subscales have high reliability and showed good convergence with comparison measures. Why was the PFRS not included in the authors' advice? To shed some light on the underlying scientific process that has ultimately led to their recommendations, I would like to ask the authors to make the workshop summaries and the focus group reports available.

Strictly speaking, the CIS, the Krupp Fatigue Severity Scale and the Chalder Fatigue Scale are all able to discriminate between CFS subjects and healthy subjects. Thus all three might indeed be used to improve the precision of CFS case ascertainment for research studies. However, if one really wishes to take CFS research forwards instead of three steps backwards, then it would be wise to abandon these low ceiling fatigue rating scales and start focussing on instruments that accurately represent the severe fatigue that is currently defined to be so characteristic for CFS.

## Competing interests

The author(s) declares that he has no competing interests.

## Authors' contributions

BS wrote the paper and performed the analysis.

## Pre-publication history

The pre-publication history for this paper can be accessed here:


